# Development and validation of a clinical decision support system based on PSA, microRNAs, and MRI for the detection of prostate cancer

**DOI:** 10.1007/s00330-023-10542-1

**Published:** 2024-01-04

**Authors:** Simone Mazzetti, Arianna Defeudis, Giulia Nicoletti, Giovanna Chiorino, Stefano De Luca, Riccardo Faletti, Marco Gatti, Paolo Gontero, Matteo Manfredi, Maurizia Mello-Grand, Caterina Peraldo-Neia, Andrea Zitella, Francesco Porpiglia, Daniele Regge, Valentina Giannini

**Affiliations:** 1 Radiology Unit, Candiolo Cancer Institute, FPO-IRCCS, Candiolo, Italy; 2 Department of Surgical Sciences, University of Turin, Turin, Italy; 3 Department of Electronics and Telecommunications, Polytechnic of Turin, Turin, Italy; 4 Cancer Genomics Lab, Fondazione Edo ed Elvo Tempia, Biella, Italy; 5 Department of Urology, San Luigi Gonzaga Hospital, University of Turin, Orbassano, Italy; 6 Radiology Unit, Department of Surgical Sciences, University of Turin, Turin, Italy; 7 Division of Urology, Department of Surgical Sciences, University of Turin, Turin, Italy

**Keywords:** Prostate cancer, Magnetic resonance imaging, microRNA, Detection, Clinical decision support system

## Abstract

**Objectives:**

The aims of this study are to develop and validate a clinical decision support system based on demographics, prostate-specific antigen (PSA), microRNA (miRNA), and MRI for the detection of prostate cancer (PCa) and clinical significant (cs) PCa, and to assess if this system performs better compared to MRI alone.

**Methods:**

This retrospective, multicenter, observational study included 222 patients (mean age 66, range 46-75 years) who underwent prostate MRI, miRNA (let-7a-5p and miR-103a-3p) assessment, and biopsy. Monoparametric and multiparametric models including age, PSA, miRNA, and MRI outcome were trained on 65% of the data and then validated on the remaining 35% to predict both PCa (any Gleason grade [GG]) and csPCa (GG ≥ 2 vs GG = 1/negative). Accuracy, sensitivity, specificity, positive and negative predictive value (NPV), and area under the receiver operating characteristic curve were calculated.

**Results:**

MRI outcome was the best predictor in the monoparametric model for both detection of PCa, with sensitivity of 90% (95%CI 73–98%) and NPV of 93% (95%CI 82–98%), and for csPCa identification, with sensitivity of 91% (95%CI 72–99%) and NPV of 95% (95%CI 84–99%). Sensitivity and NPV of PSA + miRNA for the detection of csPCa were not statistically different from the other models including MRI alone.

**Conclusion:**

MRI stand-alone yielded the best prediction models for both PCa and csPCa detection in biopsy-naïve patients. The use of miRNAs let-7a-5p and miR-103a-3p did not improve classification performances compared to MRI stand-alone results.

**Clinical relevance statement:**

The use of miRNA (let-7a-5p and miR-103a-3p), PSA, and MRI in a clinical decision support system (CDSS) does not improve MRI stand-alone performance in the detection of PCa and csPCa.

**Key Points:**

• *Clinical decision support systems including MRI improve the detection of both prostate cancer and clinically significant prostate cancer with respect to PSA test and/or microRNA.*

• *The use of miRNAs let-7a-5p and miR-103a-3p did not significantly improve MRI stand-alone performance.*

• *Results of this study were in line with previous works on MRI and microRNA.*

**Supplementary Information:**

The online version contains supplementary material available at 10.1007/s00330-023-10542-1.

## Introduction

Prostate cancer (PCa) is the most common solid cancer in men and the third most lethal in Western countries [1, 2]. Since 2020, both the European Association of Urology (EAU) and the American Urological Association (AUA) Guidelines [3, 4] strongly recommend the use of multiparametric (mp) magnetic resonance imaging (MRI) for prostate cancer diagnosis. Using this new paradigm, biopsy is spared in approximately one-third of men. Moreover, performing MRI as a triage test allows the detection of a higher rate of clinically significant (cs) PCas, while reducing the number of detected clinically insignificant lesions, limiting overdiagnosis and overtreatment [5–8]. Unfortunately, 15–20% of csPCas remain undetected with MRI, including a proportion of stromal cancers and intraductal carcinomas with a cribriform architecture [9]. Moreover, the detection of PCa on MRI is mostly based on visual and qualitative Prostate Imaging-Reporting and Data System (PI-RADS) assessment, which is a time-consuming and reader-dependent reporting process [10].

To improve the selection of patients for biopsy, other tests involving the evaluation of PCa biomarkers, such as PCA3, PHI, 4K score, SelectMDx, and ConfirmMDx, have been proposed to complement prostate-specific antigen (PSA) as minimally invasive tools for PCa detection [11]. Among candidate PCa biomarkers, circulating microRNAs (miRs), which are small non-coding RNAs that negatively regulate gene expression at the post-/transcriptional level, have also been suggested. Differential miR expression profiles between tumor and normal tissues and/or in biological fluids from PCa patients and controls have been observed and can serve as biomarkers with the potential to differentiate subjects with PCa from those with benign prostatic hyperplasia [12, 13]. In previous work, we identified two candidate diagnostic circulating miRNAs, i.e., plasma let-7a-5p and miR-103a-3p, which combined with PSA can detect csPCa better than PSA alone, especially when PSA is under 4 ng/mL [14].

In this study, we hypothesized that the clinical decision-making process could be improved by integrating different pieces of information (demographical, clinical, imaging, molecular, etc.) for a given patient in order to generate quantitative case-specific advice [15]. Recently, Keck et al used miRNA expression, PSA, clinical data, and MRI to develop a clinical decision support system (CDSS) aimed at enhancing the detection of csPCa, which yielded a sensitivity and specificity of 71.7% and 58.3%, respectively [12]. These results are not sufficient for the model to be applied in clinical practice.

The purpose of this study is to develop and validate a CDSS that includes demographics, PSA, circulating miRNAs, and MRI and to assess if such a system can improve both detection of PCa and csPCa, compared to MRI alone.

## Methods

### Study design and patients

Patients in this study were enrolled from two different prospective trials: the first in a comprehensive cancer center (center A: Candiolo Cancer Institute) from 2018 to 2020, and the second in a tertiary care hospital (center B: AOU Città della Salute e della Scienza di Torino) from January 2015 to December 2016.

All patients were referred for an MRI examination by the urologist before planning a biopsy, since they had abnormal PSA values. Inclusion criteria were as follows: (i) age ≤ 75; (ii) biopsy-naïve men; (iii) PSA ≤ 15 ng/mL; (iv) negative digital rectal examination; (v) MRI examination of the prostate, including at least axial T2-weighted (T2w) and diffusion-weighted imaging (DWI); (vi) availability of microRNA let-7a-5p and miR-103a-3p expression data; (vii) prostate biopsy within a year from MRI; (viii) written informed consent. Exclusion criteria were the following: (i) patients with a previous history of PCa and (ii) patients with contraindications to MRI.

This study was performed in accordance with the Declaration of Helsinki and the International Conference on Harmonization and Good Clinical Practice guidelines. The Ethical Committees of both centers approved the trial designs. The study is a retrospective analysis of patients previously enrolled in prospective trials, and the written informed consent covered the retrospective analyses.

### MRI acquisition protocols and examination reporting

At center A, examinations were performed using a 1.5-T MRI scanner (Optima MR450w; GE Healthcare, Milwaukee, WI, USA), with two different MRI acquisition protocols. Protocol A included the following: axial T2w, axial DWI (*b*-values 0, 800 s/mm^2^) with calculated high *b*-value imaging (*b* = 1000 s/mm^2^), and axial apparent diffusion coefficient (ADC) map, using a 32-channel phased-array coil. Protocol B included the following: multiplanar T2w sequences, DWI (*b*-values 0, 800 s/mm^2^) with calculated high *b*-value imaging (*b* = 1000 s/mm^2^), ADC map, and dynamic contrast-enhanced (DCE) acquisitions with a 32-channel phased-array coil combined with an endorectal coil (Medrad). At center B, examinations were performed using a 1.5-T MRI scanner (Achieva, Philips Medical System) with a 32-channel phased-array coil without endorectal coil, including multiplanar T2w imaging, DWI (*b*-values 50, 500, 1000 s/mm^2^), calculated high *b*-value imaging (*b*-value = 1700 s/mm^2^), ADC map, and DCE acquisitions. The detailed MRI acquisition parameters for both centers are reported in Supplementary Table 1.

Two experienced radiologists (> 8 years in prostate MRI) reviewed all MRI examinations according to the PI-RADS version 2 [16]. Patients were classified either as negative (PI-RADS 1–2), positive (PI-RADS 4–5), or equivocal (PI-RADS 3). In patients without DCE acquisitions, a modified score was applied in PI-RADS 3 cases, since peripheral zone lesions could not be upgraded to a score of 3 + 1, due to the lack of information from DCE. In men with multiple lesions with different PI-RADS classifications, the highest score was considered representing the overall PI-RADS patient status.

### miRNA

Immediately before MRI examination, blood samples from enrolled men were collected in EDTA (ethylenediaminetetraacetic acid) tubes in both centers. Plasma was isolated within 1 h from collection with a standard procedure to prevent hemolysis. Blood was centrifuged at 2500 rpm (1250 g) at 4 °C for 10 min. The supernatant was transferred into new tubes and subjected to a second centrifugation step at 2500 rpm (1250 g) at 4 °C for 10 min to remove cell debris and fragments. Plasma was stored in 4.5-mL cryovials at −80 °C until transfer to the Cancer Genomics Lab. To calculate the hemolysis score, 10 μL of plasma was centrifuged at 1000 g for 5 min at room temperature and the absorbance at 385 and 414 nm was measured by a NanoDrop spectrophotometer (Thermo Fisher) using the UV-VIS program. Samples with hemolysis score < 0.057 and/or 414 nm/385 nm absorbance ratio below 2 were kept for further processing. Total RNA was isolated from plasma with the miRNeasy serum/plasma kit (Qiagen) using Exiqon protocol, with the bacteriophage MS2-RNA carrier (Merck) to promote RNA precipitation and purification on membranes. To measure microRNA levels by SYBR green qPCR (quantitative polymerase chain reaction) analysis, miRCURY LNA™ Universal RT microRNA PCR protocol (Qiagen) was followed, starting from 4 µL of total RNA, using UniSp6 spike-in as an internal control for reverse transcription (RT). BioRad CFX96 real-time PCR instrument was used to test hsa-miR-103a-3p and hsa-let7a-5p on each sample in the same 96-well plate, with 3 replicated measurements for each test, RT, and real-time negative controls for each miR. We chose these two specific microRNAs because they were identified in a previous study [14] that included a discovery phase by microarrays, a technical validation phase with RT-qPCR, as well as a validation phase by RT-qPCR on an independent cohort. Data analysis was done following the approach explained by Deng et al [17], i.e., by averaging miR threshold cycles (Ct) and calculating the difference between let-7a-5p and miR-103a-3p averaged Cts (deltamiRNA). This approach allowed for avoiding the use of not standardized normalizers for circulating miRs and made data comparable between the two centers.

### Reference standard

Histopathology findings at biopsy were the reference standard for this study. All patients underwent biopsy, either a 12-core systematic biopsy (SBx) if MRI was negative (PI-RADS 1–2) or targeted biopsy (TBx) in combination with SBx if MRI was scored PI-RADS ≥ 3. TBx was performed using the BioJet fusion system (D&K Technologies) on a maximum of two lesions, with four to six cores retrieved from each lesion [18, 19], while 12-core SBx was performed according to the Rodrìguez-Covarrubias protocol via a transrectal approach [20]. All biopsy procedures reported the spatial localization of the prostate where the cores were retrieved, to match this information with the MRI results. Two dedicated senior uropathologists examined the stained slides and then recorded PCa presence and biopsy GG. PCa was considered clinically significant if GG ≥ 2 [7, 8].

### Monoparametric and multiparametric analysis

**Fig. 1:**
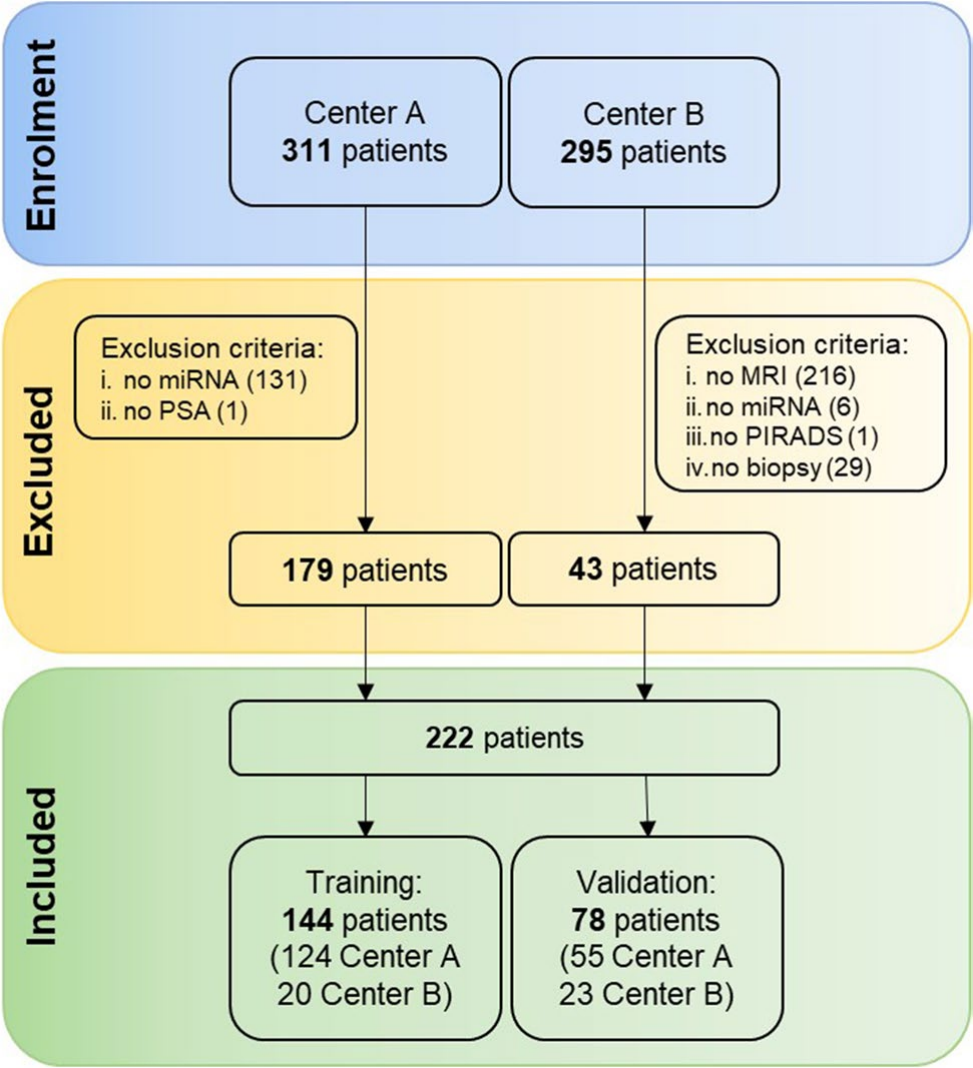
Flowchart of the study showing patients included in the analysis and composition of the training and validation datasets

Patients were randomly selected from both centers A and B to create (i) the training set for model construction (65% of patients) and (ii) the validation set for internal validation of the models (35% of the dataset, including the remaining cases). Both training and validation sets were representative of different MRI vendors, acquisition protocols, and data providers (Fig. 1).

The following clinical parameters were considered in the monoparametric and multiparametric analysis: age, PSA, deltamiRNA, and MRI outcome (either PI-RADS 1–2 or PI-RADS 3 or PI-RADS 4–5) to (a) detect PCa patients, i.e., those with biopsy-confirmed PCa (any GG) vs men with negative biopsy outcome, and (b) detect csPCa (GG ≥ 2) vs men with no csPCa (either GG = 1 or negative biopsy). PSA density was not considered in the analysis, since it is derived from MRI prostate volume, while we investigated the role of each individual examination in the prediction models.

Before analysis, all extracted features were normalized using the min–max scaling to obtain the same range of values for all parameters. In the univariate approach, the predictive value of each individual feature was computed. Then, the parameters from the univariate analysis were combined in a multivariate model whose performances were assessed using a generalized linear regression model. All algorithms were implemented using MATLAB® 2020b statistical software.

### Statistical analysis

For PCa detection, the positive class included patients predicted with PCa and the negative class those predicted with no PCa. For detection of csPCa, the positive class included patients predicted with csPCa and the negative class those predicted with non-csPCa. True positive cases were those predicted in the positive class and confirmed with either PCa or csPCa at the reference standard, while true negative cases were patients predicted in the negative class and confirmed with either no PCa or no csPCa at the reference standard. Accuracy, sensitivity, specificity, and positive and negative predictive values (PPV and NPV) were computed accordingly. Area under the receiver operating characteristic (ROC) curve (AUC) was assessed in both training/validation sets and for both univariate/multivariate analyses.

The best cutoff of the ROC curve was set as the Youden index, representing the best tradeoff between sensitivity and specificity. The cutoff obtained from the training set was applied to the validation set. Comparisons were performed using the *χ*^2^ test for categorical data, and the Mann-Whitney *U* test for continuous variables. Differences were considered statistically significant with a *p*-value < 0.05.

## Results

### Dataset

The final dataset comprised 222 patients with an average age of 66 years (range 46−75 years), including 179 men from center A and 43 from center B. Demographic, clinical, imaging, and pathological characteristics of patients and the study flowchart are presented in Table 1 and Fig. 1, respectively. Eighty patients had biopsy-confirmed PCa while 142 were negative for PCa at biopsy. Age, PSA density, and lesion volumes were not statistically different between the two centers, while PSA average values were significantly higher in center B (6.63 vs 5.71 ng/mL, *p* = 0.04).

**Table 1:** Patients’ demographics and clinical, MRI, and pathology findings. Results are presented as either counts or medians and range in parentheses

Demographics	Total	Center A	Center B	*p*-value
Patients, no.	222	179	43	
Age, year (range)	65.8 (46.1–75.0)	65.8 (46.1–74.9)	65.1 (46.4–74.4)	0.565
PSA, ng/mL (range)	5.80 (1.4–14.85)	5.71 (1.4–14.85)	6.63 (1.44–13.42)	0.037
PSA density, ng/mL/cc (range)	0.14 (0.03–0.55)	0.12 (0.03–0.55)	0.15 (0.03–0.34)	0.157
Lesion volume, cc (range)	48.0 (11.3–241)	47. 7 (11.3–241)	54.5 (20.2–103)	0.913
PI-RADS				
1–2	122 (55.0%)	107 (59.7%)	15 (34.9%)	0.003
3	21 (9.4%)	20 (11.2%)	1 (2.3%)	0.076
4–5	79 (35.6%)	52 (29.1%)	27 (62.8%)	< 0.001
Grade group (GG)				
No cancer	142 (64.0%)	114 (63.7%)	28 (65.1%)	0.883
GG 1	15 (6.8%)	12 (6.7%)	3 (7.0%)	0.944
GG 2	38 (17.1%)	31 (17.3%)	7 (16.2%)	0.888
GG 3	15 (6.8%)	13 (7.3%)	2 (4.7%)	0.557
GG 4	8 (3.6%)	6 (3.3%)	2 (4.7%)	0.657
GG 5	4 (1.8%)	3 (1.7%)	1 (2.3%)	0.792

The training set for PCa detection included 144 patients (51 with PCa and 93 with non-PCa), and the validation set included 78 patients (29 with PCa and 49 with non-PCa). The training set for the detection of csPCa included 42 patients with csPCa and 102 with non-csPCa (9 GG1 + 93 with no tumor), while the validation set was composed of 23 patients with csPCa and 55 with non-csPCa (6 GG1 + 49 with no tumor).

### Univariate analysis

**Fig. 2:**
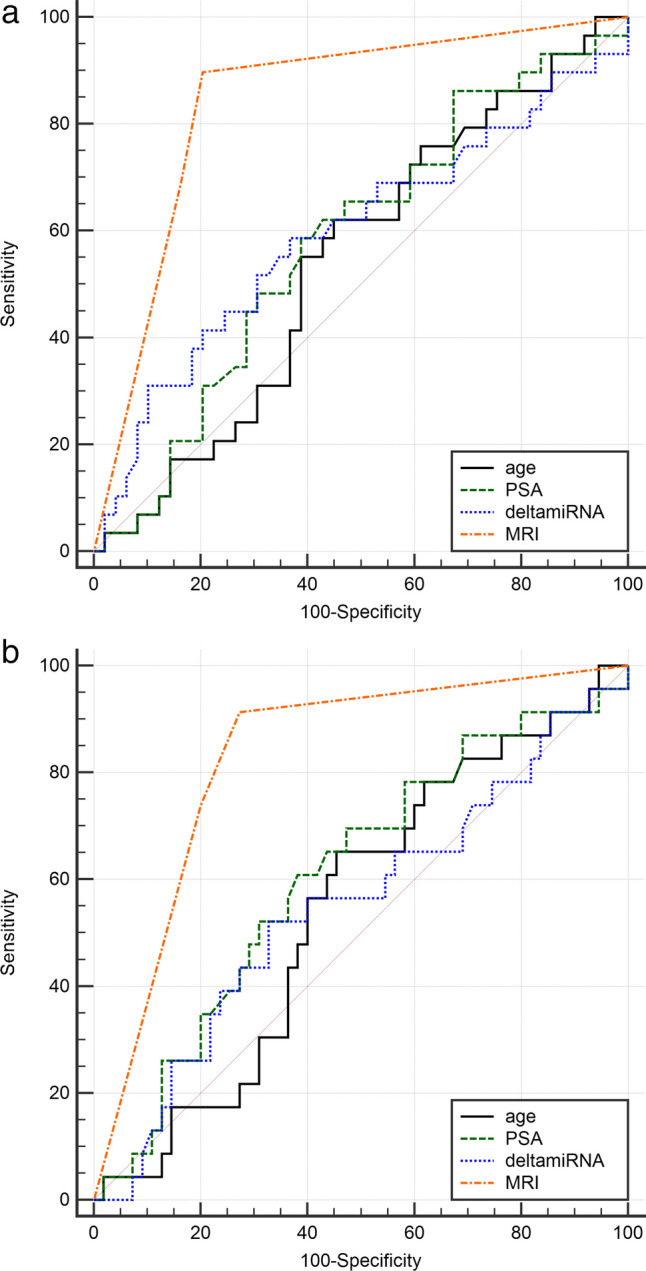
ROC curve comparison for univariate models in the **a** detection of prostate cancer and **b** detection of clinically significant prostate cancer

Table 2 shows the performances of each feature separately. The best performing parameter for PCa detection was MRI, with a sensitivity of 90%, specificity of 80%, NPV of 93%, and AUC of 0.84 on the validation set (odds ratio 33.8, 95%CI 8.48–134.67, *p* < 0.001). AUC was 0.58 for both PSA and deltamiRNA, while sensitivity on the validation set was 59% and 69%, respectively. Regarding detection of csPCa, MRI overperformed again all other variables, yielding a sensitivity of 91%, specificity of 73%, NPV of 95%, and AUC of 0.83 in the validation cohort (odds ratio 28.0, 5.84–134.2, *p* < 0.001). PSA AUC was higher than that of deltamiRNA (0.61 vs 0.54, *p* = 0.54) for the detection of csPCa, but with a lower sensitivity (61% vs 65%, *p* = 0.57), although differences were not statistically significant. The false negative (FN) cases of MRI for csPCa detection were two patients with GG2 tumors that were scored as negative for PCa by the radiologist. Using the PSA classification, there were nine FNs (six GG2, two GG3, and one GG4), while using deltamiRNA, we found eight FNs (four GG2, two GG3, and two GG4). There were no FNs in common between PSA and MRI classifiers for the detection of csPCa, while deltamiRNA had two FNs in common with MRI and three with PSA (one GG2, one GG3, and one GG4). Figure 2 shows the different AUCs for the univariate analysis in the validation set.

**Table 2:** Performances of univariate analysis for the detection of patients with suspicion of csPCa on both training and validation sets

	Training				Validation			
	Age (95% CI)	PSA (95% CI)	deltamiRNA (95% CI)	MRI (95% CI)	Age (95% CI)	PSA (95% CI)	deltamiRNA (95% CI)	MRI (95% CI)
Detection of PCa (any GG vs no tumor)								
Sensitivity	60.8% (46.1–74.2%)	64.7% (50.1–77.6%)	54.9% (40.3–68.9%)	88.2% (76.1–95.6%)	69.0% (49.2–84.7%)	58.6% (38.9–76.5%)	69.0% (49.2–84.7%)	89.7% (72.7–97.8%)
Specificity	50.5% (40.0–61.1%)	61.3% (50.6–71.2%)	43.0% (32.8–53.7%)	79.6% (70.0–87.2%)	36.7% (23.4–51.7%)	61.2% (46.2–74.8%)	36.2% (22.7–51.5%)	79.6% (65.7–89.8%)
PPV	40.3% (33.3–47.7%)	47.8% (39.8–56.0%)	34.6% (28.0–41.8%)	70.3% (61.0–78.2%)	39.2% (31.8–47.2%)	47.2% (36.0–58.8%)	40.0% (32.5–48.0%)	72.2% (59.6–82.1%)
NPV	70.2% (61.3–77.8%)	76.0% (67.9–82.6%)	63.5% (54.3–71.8%)	92.5% (85.2–96.3%)	66.7% (51.0–79.4%)	71.4% (60.6–80.3%)	65.4% (49.3–78.6%)	92.9% (81.5–97.5%)
Accuracy	54.2% (45.7–62.5%)	62.5% (54.1–70.4%)	47.2% (38.9–55.7%)	82.6% (75.5–88.4%)	48.7% (37.2–60.3%)	60.3% (48.5–71.2%)	48.7% (37.0–60.4%)	83.3% (73.2–90.8%)
AUC	0.52 (0.43–0.60) *p* = 0.685	0.62 (0.53–0.70) *p* = 0.013	0.52 (0.43–0.60) *p* = 0.770	0.85 (0.78–0.90) *p* < 0.001	0.48 (0.43–0.66) *p* = 0.510	0.58 (0.46–0.69) *p* = 0.225	0.58 (0.48–0.71) *p* = 0.177	0.84 (0.74–0.92) (*p* < 0.001)
Detection of csPCa (GG ≥ 2 vs GG = 1/no tumor)								
Sensitivity	59.5% (43.3–74.4%)	61.9% (45.6–76.4%)	57.1% (41.0–72.3%)	92.9% (80.5–98.5%)	69.6% (47.1–86.8%)	60.8% (38.5–80.3%)	65.2% (42.7–83.6%)	91.3% (72.0–98.9%)
Specificity	49.0% (39.0–59.1%)	57.8% (47.7–67.6%)	44.1% (34.3–54.3%)	75.5% (66.0–83.5%)	36.4% (23.8–50.4%)	60.0% (45.9–73.0%)	32.7% (20.7–46.7%)	72.7% (59.0–83.9%)
PPV	32.5% (26.0–39.7%)	37.7% (30.3–45.7%)	29.6% (23.5–36.6%)	60.9% (52.4–68.9%)	31.4% (24.6–39.0%)	38.9% (28.7–50.2%)	28.9% (22.2–36.5%)	58.3% (47.2–68.7%)
NPV	74.6% (66.0–81.7%)	78.7% (70.8–84.9%)	71.4% (62.4–79.1%)	96.3% (89.6–98.7%)	74.1% (58.4–85.3%)	78.6% (67.8–86.5%)	69.2% (53.4–81.6%)	95.2% (84.0–98.7%)
Accuracy	52.1% (43.6–60.5%)	59.0% (50.5–67.1%)	47.9% (39.5–56.4%)	80 6% (73.1–86.7%)	46.2% (34.8–57.8%)	60.3% (48.5–71.2%)	42.3% (31.2–54.0%)	78.2% (67.4–86.8%)
AUC	0.52 (0.44–0.61) (*p* = 0.651)	0.60 (0.52–0.68) (*p* = 0.049)	0.54 (0.45–0.62) (*p* = 0.46)	0.86 (0.79–0.91) (*p* < 0.001)	0.45 (0.43–0.66) (*p* = 0.520)	0.61 (0.49–0.72) (*p* = 0.127)	0.54 (0.43–0.66) (*p* = 0.51)	0.83 (0.73–0.91) (*p* < 0.001)

*PCa* prostate cancer, *csPCa* clinically significant prostate cancer, *GG* Gleason grade, *CI* confidence interval, *PPV* positive predictive value, *NPV* negative predictive value, *AUC* area under the receiving characteristic curve

### Multivariate analysis

Table 3 shows the results of the multiparametric analysis, considering different combinations of parameters (PSA, deltamiRNA, and MRI). The variable *age* was discarded in this analysis since its AUC was not greater than 0.5 in the validation set of the univariate analysis. All models including MRI achieved AUC in the range of 0.82–0.87 for both PCa and csPCa detection, with very high NPV (range 87.5–92.9%). The odds ratios of multivariate models with *p* < 0.05 are reported in Table 4. The PSA + deltamiRNA classifier reached statistically lower results (*p* < 0.01) than the other models including MRI, with AUC of 0.61 and 0.59 in the validation cohort for detection of PCa and csPCa, respectively. However, sensitivity and NPV of PSA + deltamiRNA were not statistically different from the other three models including MRI for the detection of csPCa (i.e., 73.9 vs 78.3%, *p* = 0.73 for sensitivity and 80.7 vs 89.4%, *p* = 0.26 for NPV).

**Table 3:** Performance of multivariate analysis for the detection of PCa and csPCa on both training and validation sets

	Training				Validation			
	PSA + MRI (95% CI)	PSA + deltmiRNA (95% CI)	MRI + deltamiRNA (95% CI)	PSA + MRI + deltamiRNA (95% CI)	PSA + MRI (95% CI)	PSA + deltmiRNA (95% CI)	MRI + deltamiRNA (95% CI)	PSA + MRI + deltamiRNA (95% CI)
Detection of PCa (any GG vs no tumor)								
Sensitivity	88.2% (76.1–95.6%)	62.8% (48.1–75.9%)	88.2% (76.1–95.6%)	88.2% (76.1–95.6%)	86.2% (68.3–96.1%)	51.7% (32.5–70.6%)	89.7% (72.7–97.8%)	89.7% (72.7–97.8%)
Specificity	80.7% (71.2–88.1%)	57.0% (46.3–67.2%)	79.6% (70.0–87.2%)	79.6% (70.0–87.2%)	81.6% (68.0–91.2%)	59.3% (44.2–73.0%)	79.6% (65.7–89.8%)	79.6% (65.7–89.8%)
PPV	71.4% (62.0–79.3%)	44.4% (36.9–52.3%)	70.3% (61.0–78.2%)	70.3% (61.0–78.2%)	73.5% (60.2–83.6%)	42.9% (31.5–55.0%)	72.2% (59.6–82.1%)	72.2% (59.6–82.1%)
NPV	92.6% (85.4–96.4%)	73.6% (65.2–80.6%)	92.5% (85.2–96.3%)	92.5% (85.2–96.3%)	90.9% (80.0–96.2%)	67.4% (57.1–76.3%)	92.9% (81.5–97.5%)	92.9% (81.5–97.5%)
Accuracy	83.3% (76.2–89.0%)	59.0% (50.5–67.1%)	82.6% (75.5–88.4%)	82.6% (75.5–88.4%)	83.3% (73.2–90.8%)	56.4% (44.7–67.6%)	83.3% (73.2–90.8%)	83.3% (73.2–90.8%)
AUC	0.86 (0.79–0.91) (*p* < 0.001)	0.62 (0.53–0.70) (*p* = 0.015)	0.87 (0.80–0.92) (*p* < 0.001)	0.87 (0.80–0.92) (*p* < 0.001)	0.82 (0.72–0.90) (*p* < 0.001)	0.61 (0.49–0.71) (*p* = 0.098)	0.87 (0.77–0.93) (*p* < 0.001)	0.84 (0.74–0.91) (*p* < 0.001)
Detection of csPCa (GG ≥ 2 vs GG = 1/no tumor)								
Sensitivity	90.5% (77.4–97.3%)	76.2% (60.6–88.0%)	88.1% (74.4–96.0%)	85.7% (71.5–94.6%)	78.3% (56.3–92.5%)	73.9% (51.6–89.8%)	78.3% (56.3–92.5%)	73.9% 51.6–89.8%
Specificity	79.4% (70.3–86.8%)	45.1% (35.2–55.3%)	78.4% (69.2–86.0%)	81.4% (72.5–88.4%)	76.4% (63.0–86.8%)	45.5% (32.0–59.5%)	74.6% (61.0–85.3%)	76.4% (63.0–86.8%)
PPV	64.4% (55.0–72.8%)	36.4% (30.9–42.2%)	62.7% (53.3–71.2%)	65.5% (55.4–74.3%)	58.1% (45.1–70.0%)	36.2% (28.7–44.4%)	56.3% (43.8–68.0%)	56.7% (43.4–69.0%)
NPV	95.3% (88.8–98.1%)	82.2% (72.0–89.2%)	94.1% (87.5–97.3%)	93.3% (86.8–96.7%)	89.4% (79.2–94.9%)	80.7% (66.4–89.8%)	89.1% (78.8–94.8%)	87.5% (77.6–93.4%)
Accuracy	82.6% (75.5–88.4%)	54.2% (45.7–62.5%)	81.3% (73.9–87.3%)	82.6% (75.5–88.4%)	76.9% (66.0–85.7%)	53.9% (42.2–65.2%)	75.6% (64.6–84.7%)	75.6% (64.6–84.7%)
AUC	0.86 (0.79–0.91) (*p* < 0.001)	0.60 (0.52–0.68) (*p* = 0.041)	0.87 (0.80–0.92) (*p* < 0.001)	0.87 (0.80–0.92) (*p* < 0.001)	0.83 (0.73–0.90) (*p* < 0.001)	0.59 (0.47–0.70) (*p* = 0.175)	0.87 (0.78–0.94) (*p* < 0.001)	0.85 (0.75–0.92) (*p* < 0.001)

*PCa* prostate cancer, *csPCa* clinically significant prostate cancer, *GG* Gleason grade, *CI* confidence interval, *PPV* positive predictive value, *NPV* negative predictive value, *AUC* area under the receiving characteristic curve

**Table 4:** Odds ratios for detection of PCa and csPCa of all multivariate models including MRI

	Odds ratio (95% CI)	*p*-value
Detection of PCa (any GG vs no tumor)		
PSA + MRI	27.78 (7.73–99.85)	< 0.0001
MRI + deltamiRNA	33.80 (8.48–134.67)	< 0.0001
PSA + MRI + deltamiRNA	33.80 (8.48–134.67)	< 0.0001
Detection of csPCa (GG ≥ 2 vs GG = 1/no tumor)		
PSA + MRI	11.63 (3.61–37.47)	< 0.0001
MRI + deltamiRNA	10.54 (3.30–33.69)	< 0.0001
PSA + MRI + deltamiRNA	9.15 (2.99–28.04)	< 0.0001

*PCa* prostate cancer, *csPCa* clinically significant prostate cancer, *GG* Gleason grade, *CI* confidence interval

**Fig. 3:**
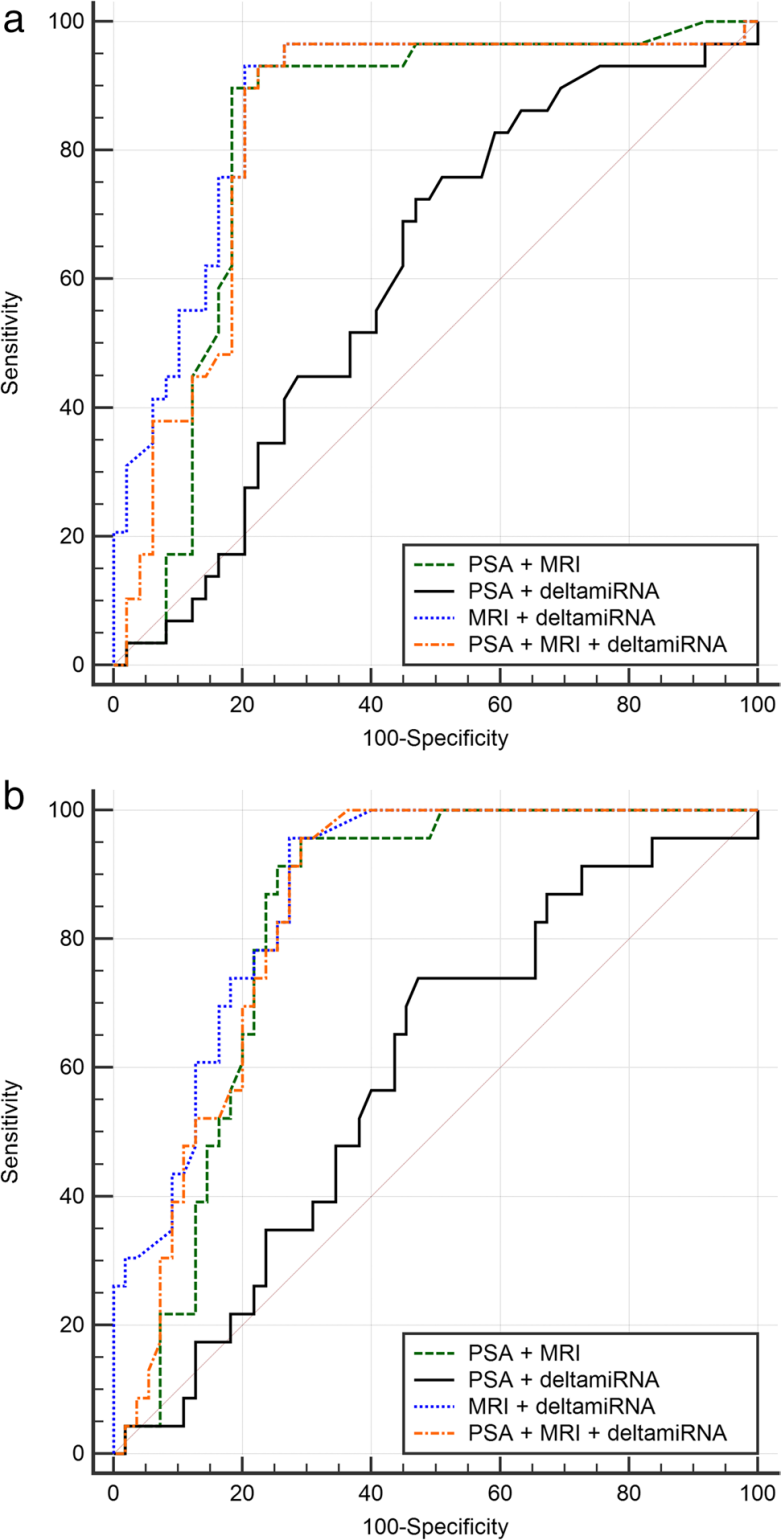
ROC curve comparison for multivariate models in the **a** detection of prostate cancer and **b** detection of clinically significant prostate cancer

**Fig. 4:**
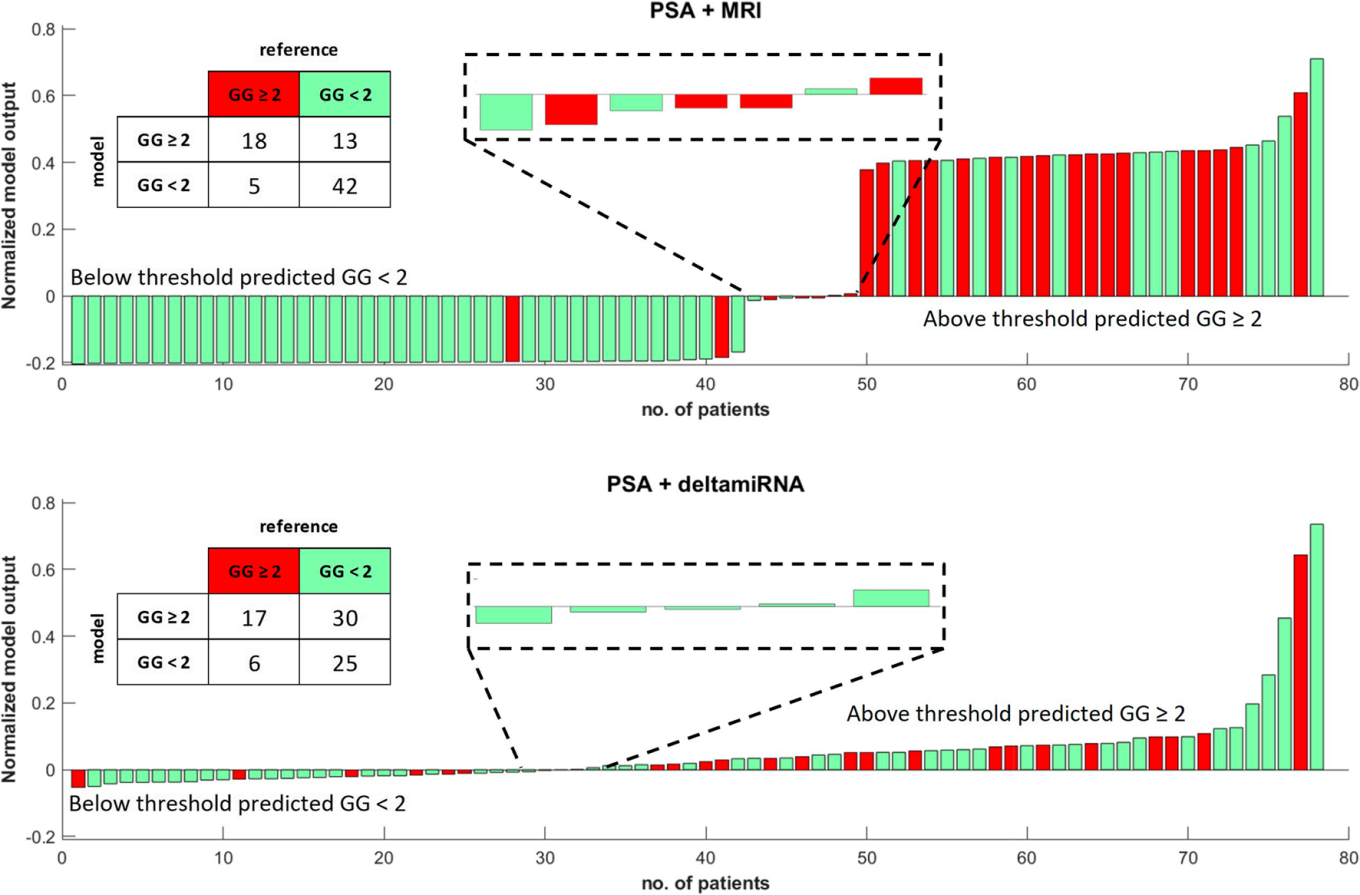
Comparison of waterfall plots and 2 × 2 tables for the detection of patients with suspicion of csPCa using PSA + MRI or PSA + deltamiRNA models in the validation set. GG gleason grade

Figure 3 compares the AUCs of the different multivariate models in the validation dataset. Figure 4 shows the waterfall plots and 2 × 2 prediction tables of PSA + MRI and PSA + deltamiRNA for the detection of csPCa in the validation set. The five FN cases of the PSA + MRI model were all GG 2 and two of them (patients nos. 28 and 41 in the upper waterfall plot) were the same FN cases in the univariate model using MRI. Among the six FN cases of the PSA + deltamiRNA model, three were GG 2, two were GG 3, and one was GG 4. Only one FN case was in common between PSA + MRI and PSA + deltamiRNA models.

## Discussion

In this study, MRI univariate analysis outperformed demographic, PSA, and deltamiRNA as a predictor of PCa presence, reaching a sensitivity of 90% and NPV of 93% on the validation set. The high NPV has important clinical implications since men with a negative MRI examination could safely avoid biopsy, with benefits for their quality of life [21–23]. MRI was also the best predictor of csPCa, compared to the other variables, with a sensitivity of 91% and NPV of 95% on the validation set.

In multivariate analysis, none of the models adding PSA to MRI or to MRI + deltamiRNA performed better than MRI alone for both PCa and csPCa detection. Previously, several studies have described prediction models using miRNAs, in particular, let-7a-5p and miR-103a-3p as in this work, to stratify PCa patients. Kong et al [24] showed that the deregulated expression of miRNAs contributes to the initiation and progression of PCa. Among several known miRNAs, they considered the let-7 family, since it appears to play a key role in the recurrence and progression of PCa by regulating cancer stem cells. In their study, they found that the expression of the let-7 family was lost in PCa tissue specimens with Gleason grade 7 or higher but not in patients with Gleason grade 6, with an inverse correlation between miRNA expressions and PCa aggressiveness (*p* < 0.05). Ge et al [25] explored the effect and potential mechanism of miR-103a-3p in PCa, showing that miR-103a-3p inhibits tumor cell proliferation, invasion, and wound healing ability of PCa, while promoting apoptosis. Other studies combined miRNAs with PSA as biomarkers for treatment decisions [12, 14], such as Mello-Grand et al who proposed a model including circulating PSA and miRNAs to detect PCa. In their work, a classifier based on let-7a-5p, miR-103a-3p, and PSA was proposed to identify csPCa better than PSA alone, even in 50–69-year-old men with PSA ≤ 4 ng/mL. Their model identified eight out of nine additional tumors undetected by PSA, including three high-risk PCa, yielding an AUC of 0.76, compared to 0.74 of the PSA [14]. In our work, we also found an improvement in AUC using the combination of PSA and deltamiRNA, instead of PSA alone for PCa detection, although the difference was not statistically significant (0.61 vs 0.58, respectively, *p* = 0.104). We hypothesize that the higher performance in their study could have been determined by the different distributions of PSA values among enrolled patients. Our dataset included 87% (193/222) of men with PSA between 4 and 16 ng/mL, compared to their 56% (202/362). Indeed, their classifier yielded an AUC of 0.6 on the validation set when only patients with PSA in the range 4–16 ng/mL were considered, and an AUC of 0.47 using the PSA alone, results that are almost equal to our PSA + deltamiRNA model.

Keck et al tested a regression model that included several miRNAs and four other variables (patient age, pre-biopsy PSA, previous biopsy procedure, and the highest MRI PI-RADS score) [12]. When the let-7c-5p miRNA was incorporated into the model, a sensitivity of 72% and specificity of 58% for the detection of PCa were reached on the validation set. Comparing these results to our multivariate model for PCa detection, we obtained better performances, with a sensitivity and specificity of 89.7% and 79.6%, respectively. Pecoraro et al have also implemented an integrated pathway based on clinical features, MRI, and miRNAs for the detection of PCa on a dataset composed of 178 patients. Their findings showed that the integrated pathway was not statistically better than the MRI alone, with reported AUC of 0.904 and 0.880, respectively [26].

In our study, the combinations PSA + deltamiRNA and PSA + MRI allowed a reduction of 33% and 44% of FN cases, respectively, compared to PSA alone, the latter yielding nine FN findings. Interestingly, the PSA + deltamiRNA model allowed the identification of three additional GG2, while the PSA + MRI model identified all GG ≥ 3 (*n* = 4).

The main strength of our work is the validation step on a dataset never used for model implementation. Previously, only a few studies validated the correlation between miRNAs and PCa detection in an independent cohort, with only Keck et al [12] including MRI and other clinical variables in their models. This step is essential to demonstrate accuracy of predictive models and their clinical relevance.

Our study has also some limitations. First, we did not assess different sets of miRNAs, as performed in other studies, because the let-7a-5p and miR-103a-3p were the only two miRNAs in common between the two datasets of this retrospective study. The combination of other miRNAs in a multivariate analysis including PSA, MRI, and other clinical variables might lead to better performances [13]. Second, our dataset included 222 patients, with only 65 patients classified with GG ≥ 2 lesions, leading to quite an unbalanced dataset. Moreover, having 222 patients over a 5-year accrual period can lead to selection bias. Indeed, the relatively small size of the dataset was mainly due to the study design and inclusion criteria, which required molecular information (let-7a-5p and miR-103a-3p expression data), MRI, and biopsy for all subjects. To reduce model overfitting, the results were validated on an independent dataset.

In conclusion, our findings confirmed that MRI stand-alone yielded the best prediction model for both detection of PCa and csPCa. Moreover, our study showed that miRNAs let-7a-5p and miR-103a-3p alone or in combination with PSA do not increase the stand-alone performance of MRI in this study population.

### Supplementary Information

Below is the link to the electronic supplementary material.

Supplementary file1 (PDF 150 KB)

## Abbreviations

ADC: Axial apparent diffusion coefficient
AUA: American Urological Association
AUC: Area under the curve
averaged Cts: deltamiRNA
CDSS: Clinical decision support system
cs: Clinically significant
Ct: Cycles
DCE: Dynamic contrast-enhanced
DWI: Diffusion-weighted imaging
EAU: European Association of Urology
EDTA: Ethylenediaminetetraacetic acid
FN: False negative
GG: Gleason grade
miRs: microRNAs
mpMRI: Multiparametric magnetic resonance imaging
NPV: Negative predictive value
PCa: Prostate cancer
PI-RADS: Prostate Imaging-Reporting and Data System
PPV: Positive predictive value
PSA: Prostate-specific antigen
qPCR: Quantitative polymerase chain reaction
ROC: Receiver operating characteristic
RT: Reverse transcription
SBx: Systematic biopsy
T2w: T2-weighted
TBx: Targeted biopsy

## Declarations

### Guarantor

The scientific guarantor of this publication is Daniele Regge.

### Statistics and biometry

One of the authors has significant statistical expertise.

### Study subjects or cohorts overlap

No study subjects or cohort overlap has been previously reported.

### Methodology

• retrospective

• observational

• multicenter study
