# Supplementary material for: Development and validation of a clinical decision support system based on PSA, microRNAs, and MRI for the detection of prostate cancer
Source: Eur Radiol. 2024 Jan 4;34(8):5108–17. doi: 10.1007/s00330-023-10542-1 (PMC11255044; doi:10.1007/s00330-023-10542-1)
Supplement: Supplementary file 1 — Supplementary file1 (PDF 150 KB) [file 330_2023_10542_MOESM1_ESM.pdf]

Supplementary Table 1: MRI acquisition protocols for Centers A and B

	Center A			Center B		
	T2w	DWI	DCE	T2w	DWI	DCE
**Field of view (mm)**	160 x 160	160 x 160	200 x 200	180 x 180	180 x 180	180 x 180
**In plane resolution (mm)**	0.36 x 0.36	2.23 x 2.23	0.71 x 0.71	0.38 x 0.38	1.88 x 1.88	0.94 x 0.94
**section thickness/ gap (mm)**	3/0	3/0	3/0	3/0	3/0	3/0
**Repetition time (ms)**	3020	7000	3.6	5403	2919	4.7
**Echo time (ms)**	85	101	1.3	100	61	2.2
**Flip angle (degrees)**	160	90	20	90	90	10
**b-values (s/mm^2)**	-	0, 800; 1000 (calculated)	-	-	50, 500, 1000; 1700 (calculated)	-

T2w: T2weighted image*, DWI:* Diffusion-weighted *imaging, DCE: Dynamic Contrast Enhanced*
